# Factors Associated With Quality Care Among Adults With Rheumatoid Arthritis

**DOI:** 10.1001/jamanetworkopen.2022.46299

**Published:** 2022-12-12

**Authors:** Anne V. Seyferth, Meghan N. Cichocki, Chien-Wei Wang, Yun-Ju Huang, Yi-Wei Huang, Jung-Sheng Chen, Chang-Fu Kuo, Kevin C. Chung

**Affiliations:** 1 Section of Plastic Surgery, Department of Surgery, University of Michigan Medical School, Ann Arbor; 2 School of Medicine, Chang Gung University, Taoyuan, Taiwan; 3 Division of Rheumatology, Allergy and Immunology, Chang Gung Memorial Hospital, Taipei, Taiwan; 4 Center for Artificial Intelligence in Medicine, Chang Gung Memorial Hospital, Taoyuan, Taiwan

## Abstract

**Importance:**

Although quality care markers exist for patients with rheumatoid arthritis (RA), the predictors of meeting these markers are unclear.

**Objective:**

To explore factors associated with quality care among patients with RA.

**Design, Setting, and Participants:**

A retrospective cohort study using insurance claims from 2009 to 2017 was conducted, and 6 sequential logistic regression models were built to evaluate quality care markers. Quality care markers were measured at 1 year post-RA diagnosis for each patient. The MarketScan Research Database, which contains commercial and Medicare Advantage administrative claims data from more than 100 million individuals in the US, was used to identify patients aged 18 to 64 years with a diagnosis claim for RA. Patients with conditions presenting similar to RA and missing demographic characteristics were excluded. Data analysis occurred between February 18 and May 5, 2022.

**Exposures:**

Success or failure to meet selected RA quality care markers within 1 year after RA diagnosis.

**Main Outcomes and Measures:**

Prevalence of meeting successive quality care markers for RA.

**Results:**

Among 581 770 patients, 430 843 (74.1%) were women and the mean (SD) age was 48.9 (11.3) years. Most patients (236 285 [40.6%]) resided in the South and had an income less than or equal to $45 200 (490 366 [84.3%]). Of the total study population, 399 862 individuals (68.7%) met at least 1 quality care marker and 181 908 (31.3%) met 0 markers. Most commonly, patients met annual laboratory testing (299 323 [51.5%]) and referral to a rheumatologist (256 765 [44.1%]) markers. The least met marker was receiving hepatitis B screening prior to initiation of disease-modifying antirheumatic drug (DMARD) therapy (18 548 [3.2%]). Women were most likely to meet all quality care markers except receiving DMARDs with hepatitis B screening (odds ratio, 1.14; 95% CI, 1.12-1.16). Individuals with lower median household income had lower odds of receiving a rheumatologist referral, an annual physical examination, or annual laboratory testing, but greater odds of receiving the other quality care markers. Patients with Medicare and those with comorbidities were generally less likely to meet quality care markers.

**Conclusions and Relevance:**

In this cohort study of patients with RA, findings indicated downstream associations with rheumatologist referral and receiving DMARDs and varied associations between meeting quality care markers and patient characteristics. These findings suggest that prioritizing early care, especially for vulnerable patients, will ensure that quality care continues.

## Key Points

**Question:**

What factors are associated with achievement of quality care markers among patients with rheumatoid arthritis?

**Findings:**

In this cohort study including data from 581 770 individuals, patients who were male, had Medicare insurance, or had increased comorbidities were generally less likely to meet the selected rheumatoid arthritis quality care markers. Receiving a rheumatologist referral and disease-modifying antirheumatic drugs was associated individually with meeting additional downstream quality care measures.

**Meaning:**

The findings of this study suggest that quality care for patients with rheumatoid arthritis can be improved by prioritizing early treatment and focusing on vulnerable patient populations.

## Teaser

This cohort study assesses the prevalence of meeting successive markers of quality care for individuals with rheumatoid arthritis and characteristics associated with meeting these markers.

## Introduction

Rheumatoid arthritis (RA) is a chronic, progressive disease that causes severe joint pain and irreversible deformities, leading to a diminished quality of life. Uncontrolled RA can also lead to substantial morbidities and mortalities secondary to its extra-articular manifestations.^1^ Rheumatoid arthritis affects an estimated 1.3 million adults in the US and is associated with an annual health care cost of $19.3 billion.^2,3^

Despite advances in novel biologic therapies and evidence on early initiation of these disease-modifying antirheumatic drugs (DMARDs), quality of care for RA remains substandard.^4,5,6^ There is poor adherence to guidelines, including prescribing DMARDs.^4,7^ Variations in care by geographic region and physician specialty indicate areas for improvement.^8^ Various quality care markers have been developed to establish a standard of care and assess outcomes for RA.^6,8^ Studying these markers can aid in understanding treatment variations and factors affecting nonadherence. Closing these gaps may help improve outcomes and decrease financial burdens on patients and health care systems.

We used 6 quality care measures for RA identified by the Arthritis Foundation, a national organization for RA care^9^: referral to a rheumatologist, hepatitis B screening prior to DMARD initiation, baseline hand radiographs within 1 year of the initial visit, annual physical examination, annual laboratory testing, and physical therapy (PT), occupational therapy (OT), or hand surgery referral. We sought to understand the interplay between each selected quality care marker for RA and demographic variability. By assessing the status of achieving quality measures at 1 year post-RA diagnosis, we hypothesized that failure at one quality care marker is associated with greater likelihood of failure at other markers and that certain demographic factors, such as sex and income, are associated with decreased adherence.

## Methods

### Data Source and Cohort Selection

**Figure 1.**
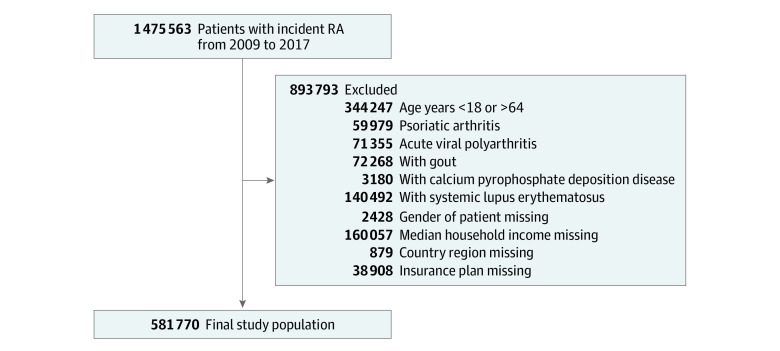
Flow Diagram of Study Inclusion Criteria RA indicates rheumatoid arthritis.

This retrospective observational cohort study used IBM Truven MarketScan Research Database insurance claims data from 2009 to 2017.^10^ Data analysis occurred between February 18 and May 5, 2022. The study cohort included adults with RA aged 18 to 64 years at first diagnosis (index date), identified by *International Classification of Diseases, Ninth Revision, Clinical Modification* (*ICD-9-CM*) codes and *International Statistical Classification of Diseases, Tenth Revision* (*ICD-10*) codes. Codes are available in eTable 1 in Supplement 1. We included a 1-year preindex look-back period (or to the earliest date of the claim for patients in 2009) to ensure no prior RA diagnosis existed and a 1-year follow-up period. We excluded patients with inflammatory conditions clinically similar to RA (Figure 1). Because of the different etiology of RA, patients with arthritis due to physical use, such as osteoarthritis, were not excluded. The University of Michigan exempted this study from institutional review board review because the data were deidentified. This study followed the Strengthening the Reporting of Observational Studies in Epidemiology (STROBE) reporting guideline for observational studies.

### Variables of Interest

In 2004, the Arthritis Foundation developed 27 quality markers for RA, representing a minimally acceptable standard of care. These markers were developed by a clinical committee, literature review, and expert panel, and have been reviewed for scientific evidence.^8,9^ Our selection criteria were limited to markers suitable for analysis using insurance claims data. First, several markers require patient-reported data, such as difficulty performing tasks. These reports cannot be identified in claims data and were excluded from our analysis. Second, we chose measures applicable to most patients with RA, modeling a realistic clinical experience of patients. For example, we included rheumatologist referral and excluded markers related to reproductive issues. We recognize the importance of studying all quality measures but sought to understand RA care within the limitations of our database. Where applicable, we adjusted markers to align with updated evidence. Recent guidelines recommend DMARD therapy initiation as soon as RA is diagnosed and to include hepatitis B screening; we used this adjusted guideline.^11,12^ Our final selected quality care markers and order for the logistic regression models were referral to a rheumatologist, hepatitis B screening prior to DMARD initiation, baseline hand radiographs, annual physical examination, annual laboratory testing, and PT, OT, or hand surgery referral. The Arthritis Foundation recommends many of the quality measures be met within 3 months of the index diagnosis; we evaluated each quality measure 1 year postindex to account for potential delays in care. In addition, to assess for potential confounders, variables in model 1 were tested with univariate analysis; all variables were found to be statistically significant (*P* < .001), indicating that each explanatory variable is statistically associated with outcome. The included markers were predetermined based on rheumatologist and hand surgeon clinical experiences. We considered the Elixhauser Comorbidity Index in the model to evaluate the overall comorbidity burden and the Rheumatic Disease Comorbidity Index (RDCI), a validated measure for rheumatic disease, in the model to consider the RA-related outcomes of death and physical functioning.^13^ We believe the inclusion of both tools maximizes the available information from the database relevant to the response variable. Additional confounders may still exist, such as race and ethnicity or educational level; however, these variables are not included in the MarketScan database and therefore were not included in the models.

To investigate demographic characteristics associated with achieving quality care, we considered sex, age at index, geographic region of residence, median household income, insurance source (commercial or Medicare), insurance plan (comprehensive or other), and comorbidities. We linked patients’ metropolitan statistical area to the 2010 census data to obtain the median household income. We used *ICD-9-CM* and *ICD-10* codes to identify the Elixhauser Comorbidity Index score for each patient.^14,15^ The RDCI was used to quantify the comorbidity burden.^13,16^ Heart diseases, diabetes, and gastrointestinal bleeds were investigated individually because of their association with RA.^17,18,19^

### Model Design and Statistical Analysis

We constructed 6 logistic regression models using the chosen quality care markers. Each model contains all independent covariates and sequentially adds 1 quality care marker. Thus, the model for receiving DMARDs with hepatitis B screening included an marker for referral to a rheumatologist; the model for receiving a hand radiograph included both referral to a rheumatologist and DMARDs with hepatitis B screening, and so on.^20^ This design and the predetermined order of models were chosen to evaluate a hypothetical yet realistic care pathway and investigate whether success at earlier markers is associated with success at later markers.^21^ Analyses used a 2-sided, unpaired significance level set at *P* < .05. Data management and analyses were performed with SAS, version 9.4 software (SAS Institute Inc).

## Results

Using data between 2009 and 2017, we identified 1 475 563 patients with RA. After applying inclusion and exclusion criteria, our study cohort was 581 770 patients, with 430 843 women (74.1%) and 150 927 men (25.9%); mean (SD) age was 48.9 (11.3) years (Figure 1). Most patients (236 285 [40.6%]) resided in the South and had an income above $45 200 (490 366 [84.3%]). A total of 316 180 patients (54.3%) did not have any comorbidities in the Elixhauser Comorbidity Index at diagnosis; 265 590 (45.7%) patients had an Elixhauser Comorbidity Index score greater than or equal to 1. A total of 155 489 (26.7%) patients had an RDCI score of 0; 221 899 (38.1%) patients had an RDCI score of 1 to 2, and 204 382 (35.1%) patients scored greater than 2. Other comorbidities included heart disease (160 752 [27.6%]), diabetes (116 489 [20.0%]), and gastrointestinal bleeds (175 801 [30.2%]). Baseline patient characteristics are presented in the Table.

**Table.** Characteristics of the Study Population

Variable	No. (%)
	Total (n = 581 770)
Age at index date, mean (SD), y	48.9 (11.3)
Age group, y	
18-24	22 315 (3.8)
25-34	53 954 (9.3)
35-44	102 890 (17.7)
45-54	180 388 (31.0)
55-64	222 223 (38.2)
Sex	
Female	430 843 (74.1)
Male	150 927 (25.9)
Country region	
West	100 288 (17.2)
North Central	119 028 (20.5)
Northeast	126 169 (21.7)
South	236 285 (40.6)
Median household income, $	
<45 200	91 404 (15.7)
>45 200	490 366 (84.3)
Type of insurance plan	
Comprehensive	12 853 (2.2)
Other	568 917 (97.8)
Insurance source (commercial or Medicare)	
Commercial	579 276 (99.6)
Medicare	2494 (0.4)
Referral to a rheumatologist	256 765 (44.1)
DMARD prescription	
None	392 589 (67.5)
Biologic DMARDs	20 182 (3.5)
Conventional DMARDs	127 168 (21.9)
Targeted synthetic DMARDs	472 (0.1)
Biologic DMARDs + conventional DMARDs	40 180 (6.9)
Biologic DMARDs + targeted synthetic DMARDs	145 (0.0)
Conventional DMARDs + targeted synthetic DMARDs	674 (0.1)
Biologic DMARDs + conventional DMARDs + targeted synthetic DMARDs	360 (0.1)
Hepatitis B screening	34 743 (6.0)
Hand radiographs	55 817 (9.6)
Physical therapy referral	4203 (0.7)
Occupational therapy referral	1567 (0.3)
Hand surgery referral	17 690 (3.0)
Annual physical examination	24 223 (4.2)
Annual laboratory work	299 323 (51.5)
Comorbidity index	
Elixhauser	
0	316 180 (54.3)
1-3	52 278 (9.0)
4-8	122 202 (21.0)
>8	91 110 (15.7)
RDCI	
0	155 489 (26.7)
1-2	221 899 (38.1)
>2	204 382 (35.1)
Heart diseases	160 752 (27.6)
Diabetes	116 489 (20.0)
Gastrointestinal bleeds	175 801 (30.2)
Laboratory testing within 1 y of the initial visit or diagnosis	124 324 (21.4)

Abbreviations: DMARDs, disease-modifying antirheumatic drugs; RDCI, Rheumatic Disease Comorbidity Index.

Of the total study population, 399 862 individuals (68.7%) met at least 1 quality care marker and 181 908 patients (31.3%) did not meet any quality care markers. The percentages of patients who met each marker were referral to a rheumatologist (256 765 [44.1%]), DMARDs with hepatitis B screening (18 548 [3.2%]), hand radiographs (55 817 [9.6%]), annual physical examination (24 223 [4.2%]), annual laboratory testing (299 323 [51.5%]), and PT, OT, or hand surgery referral (22 227 [3.8%]). Odds ratios and 95% CIs for each model are available in eTable 2 in Supplement 1.

### Model 1: Referral to Rheumatologist

**Figure 2.**
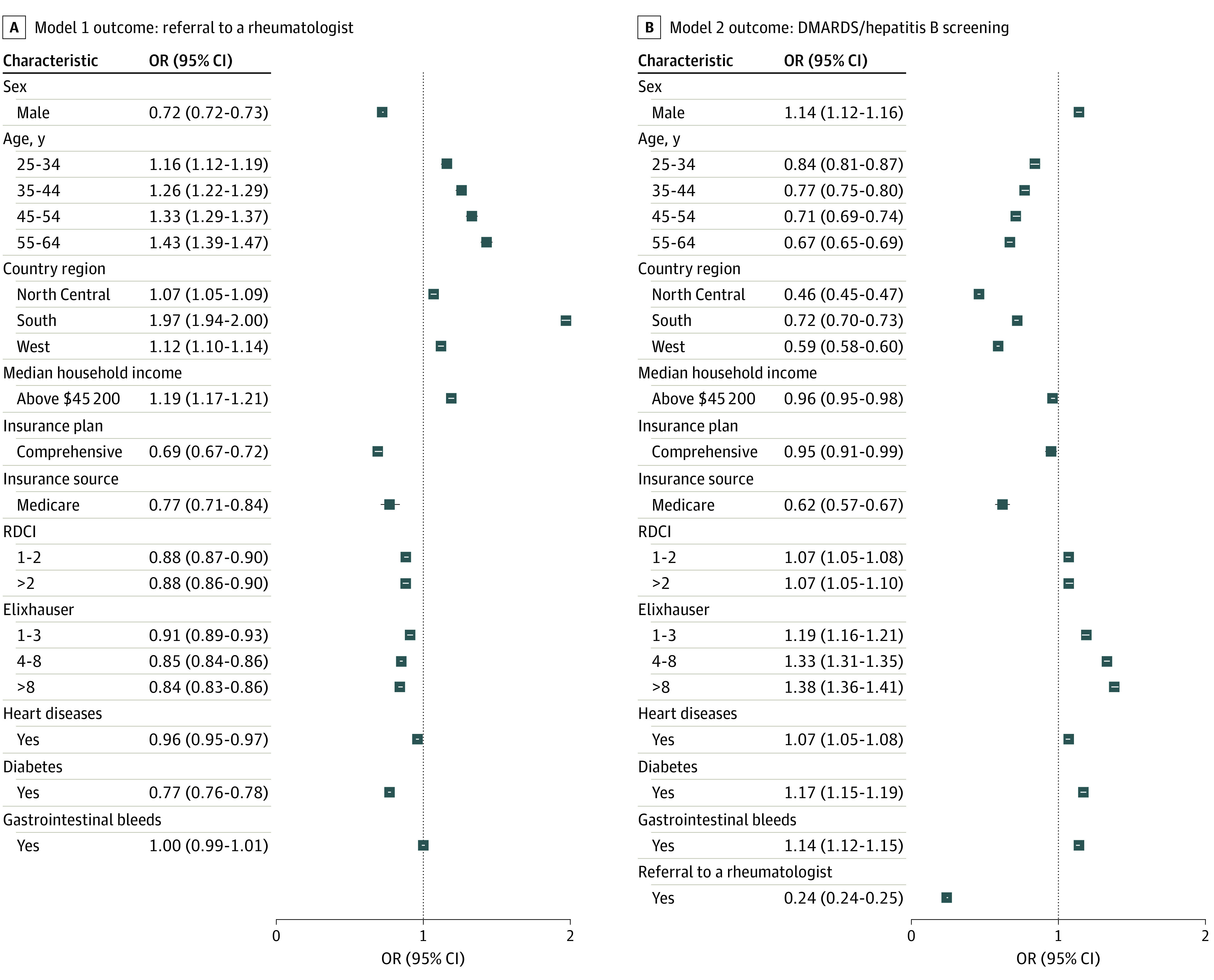
Odds of Outcomes With Models 1 and 2 A, Model 1: referral to rheumatologist. B, Model 2: prescribing of disease-modifying antirheumatic drugs (DMARDs) with hepatitis B screening. OR indicates odds ratio; RDCI, Rheumatic Disease Comorbidity Index.

Men, patients from the Northeast, and those with lower income (<$45 200) had decreased odds of seeing a rheumatologist (Figure 2A). There was an age gradient in which younger patients had lower odds of receiving a referral than older patients. Patients with comprehensive or Medicare insurance were at lower odds of a referral to a rheumatologist. Elixhauser Comorbidity Index and RCDI scores greater than 0 were associated with decreased odds of referral.

### Model 2: DMARDs With Hepatitis B Screening

Within 1 year of RA diagnosis, 32.5% of patients (189 181) received DMARDs; 34 743 (6.0%) had prior hepatitis B screening (Table). Patients with a rheumatologist referral had significantly decreased odds of receiving a prescription for DMARDs with hepatitis B screening (Figure 2). Women; patients older than 24 years; those residing in the North Central, South, or West regions; and patients with a higher income had decreased odds of receiving DMARDs with hepatitis B screening. Patients without comorbidities (0 on the Elixhauser Comorbidity Index and the RDCI score and without heart disease, diabetes, or gastrointestinal bleeds) had lower odds of receiving DMARDs with hepatitis B screening.

### Model 3: Hand Radiographs Within 1 Year

**Figure 3.**
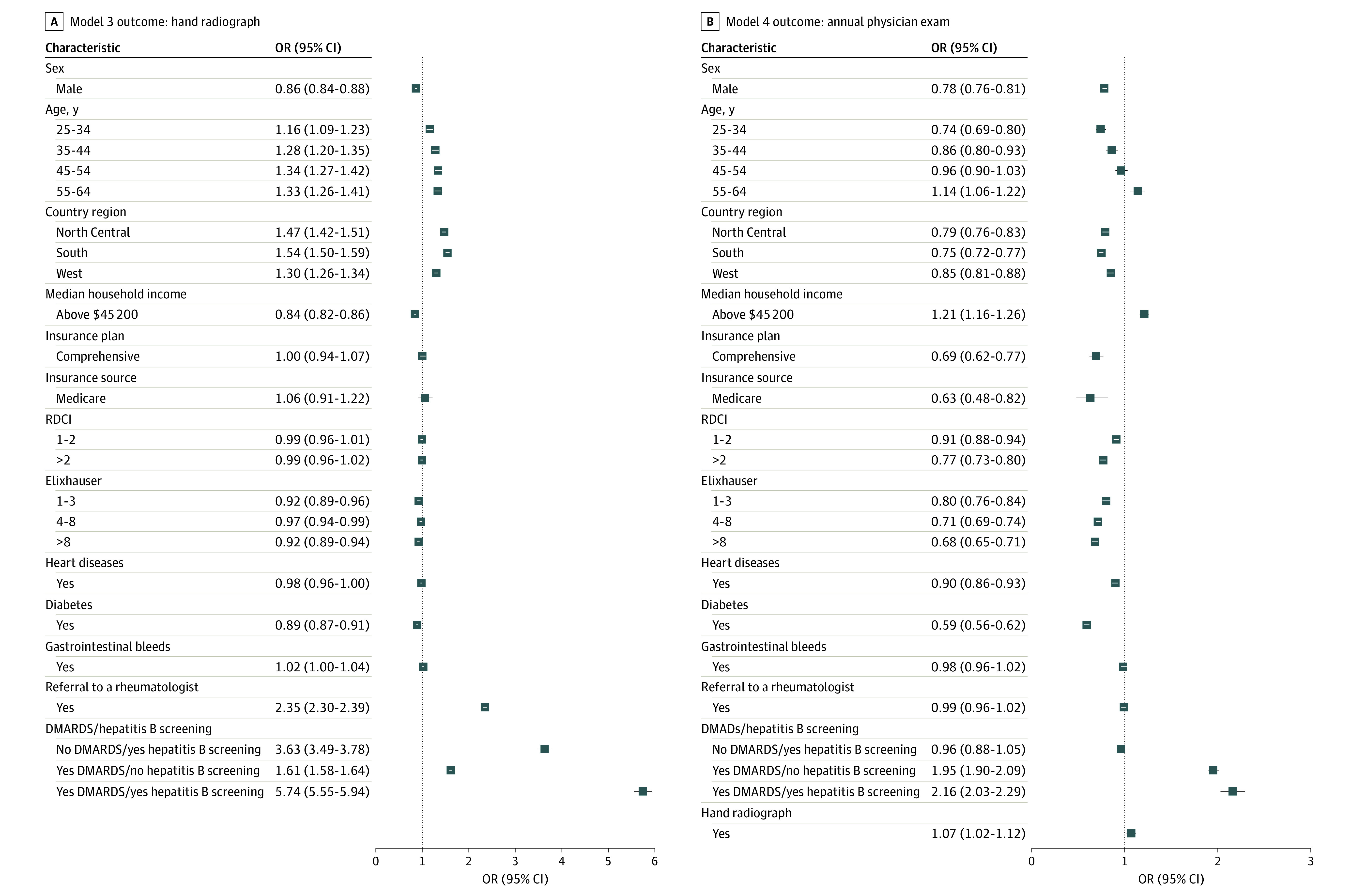
Odds of Outcomes With Models 3 and 4 A, Model 3: hand radiographs within 1 year. B, Model 4: annual physical examination. DMARDS indicates disease-modifying antirheumatic drugs; OR, odds ratio; RDCI, Rheumatic Disease Comorbidity Index.

A total of 55 817 patients (9.6%) received a hand radiograph at 1 year postindex (Table). Patients who lacked a rheumatologist referral and those who did not receive DMARDs with hepatitis B screening had decreased odds of receiving a hand radiograph (Figure 3). Men, patients with a higher income, and those residing in the Northeast were less likely to have a hand radiograph. A slight age gradient existed whereby younger patients were associated with decreased odds of receiving a radiograph.

### Model 4: Annual Physical Examination

Within 1 year of diagnosis, 24 223 (4.2%) of patients received a physical examination (Table). Failure to receive hand radiographs, DMARDs with hepatitis B screening, or DMARDs alone independently decreased the odds of receiving an annual physical examination (Figure 3). Men; patients from the North Central, South, or West regions; and those with lower income had lower odds of receiving an examination. Age showed a nonuniform gradient effect: adults aged 25 to 34 and 35 to 44 years were less likely to receive an annual physical examination, whereas adults aged 55 to 64 years were more likely. Having Medicare or comprehensive insurance was associated with decreased odds of receiving an examination. Furthermore, patients with comorbidities (Elixhauser Comorbidity Index or RDCI score >0 and with heart disease or diabetes) had decreased odds of having an annual physical examination.

### Model 5: Annual Laboratory Testing

**Figure 4.**
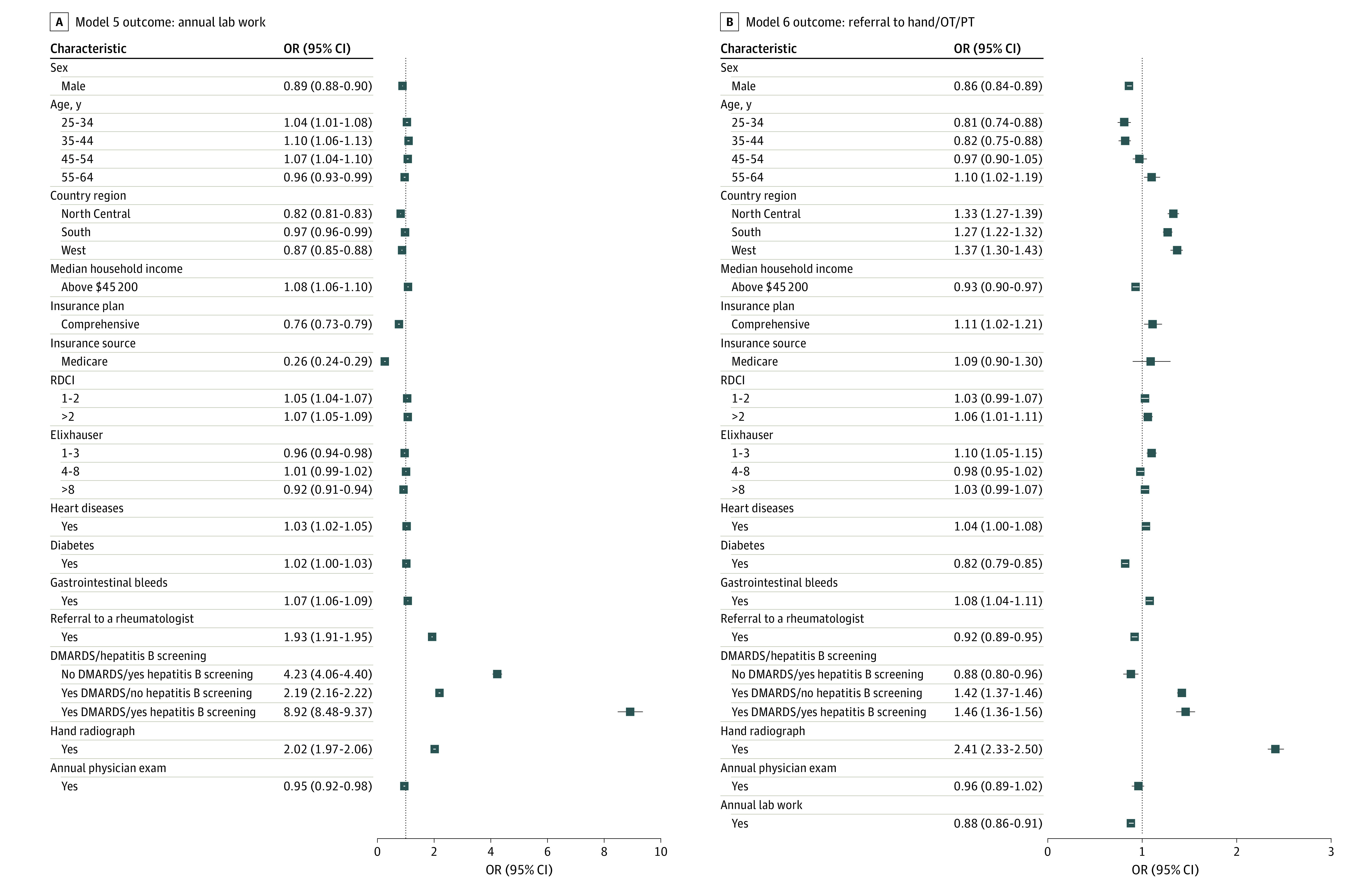
Odds of Outcomes With Models 5 and 6 A, Model 5: annual laboratory testing. B, Model 6: referral to hand surgery, occupational therapy (OT), and physical therapy (PT). DMARDS indicates disease-modifying antirheumatic drugs; OR, odds ratio; RDCI, Rheumatic Disease Comorbidity Index.

Within 1 year of RA diagnosis, 124 324 patients (21.4%) received laboratory testing (Table). Failure to receive a rheumatologist referral or hand radiographs was associated with lower odds of obtaining laboratory testing (Figure 4). An annual physical examination was also associated with lower odds. Patients who did not receive DMARDs with hepatitis B screening had decreased odds of receiving annual laboratory testing. Men and patients with Medicare or comprehensive insurance also had lower odds of receiving laboratory testing. Results for other covariates were variable.

### Model 6: Referral for Hand Surgery, OT, or PT

A total of 4203 patients (0.7%) were referred to PT, 1567 patients (0.3%) were referred to OT, and 17 690 patients (3.0%) received a hand surgery referral within 1 year from their RA index date (Table). Patients with a rheumatologist referral and annual laboratory testing had independently decreased odds of a referral to hand surgery, PT, or OT (Figure 4). Independently, patients who received hand radiograph, those who received DMARDs but no hepatitis B screening, or both DMARDs and hepatitis B screening had increased odds of a referral. Men, patients with a higher income, and those with noncomprehensive insurance had lower odds of a hand surgery, PT, or OT referral. Comorbidities produced varied effects: diabetes decreased the odds and gastrointestinal bleeds increased the odds of receiving a referral.

## Discussion

Early diagnosis and treatment of RA are associated with better outcomes including lower rates of radiographic progression and development of disability.^22^ Despite widespread evidence supporting early treatment, the quality of health care for patients with RA is suboptimal.^5^ Using select quality care markers, our study aimed to identify the prevalence of missed markers based on patient demographic data and examine whether these markers have an association with other markers. Our study found downstream associations of some RA quality care markers, including rheumatologist referral and receiving DMARDs. Notable variation existed among demographic characteristics associated with achieving quality care measures. In addition, only 27 patients of the 581 770 included in the cohort met all care markers; there is an urgent need to improve the progression of care for patients with RA.

The American College of Rheumatology and the Arthritis Foundation recommend that patients should be referred to a rheumatologist for diagnostic evaluation and management of inflammatory arthritis.^9,23^ Despite this, only 44.1% of patients in our study received a referral to a rheumatologist within 1 year of diagnosis. This time was longer but consistent with previous literature, which has reported average delays of 115 days between initial presentation and rheumatologist referral.^24,25^ Receiving a rheumatologist referral had variable associations with meeting other quality care markers, conferring greater odds of receiving hand radiographs and laboratory testing but lower odds of receiving DMARDs with appropriate hepatitis B screening or a PT, OT, or hand surgery referral. Because early RA treatment estimates improved outcomes,^22^ this negative finding may result from inappropriate timing of DMARD initiation or surgery if a patient is referred long after symptom onset. DMARDs can slow RA progression and improve long-term disability; however, treatment must be started early in symptom onset (within 12 weeks) to obtain maximal protection.^26^ In addition, lack of agreement and collaboration between rheumatologists and hand surgeons may prevent patients from receiving a hand surgery referral after seeing a rheumatologist.^27^ The findings related to receiving a rheumatologist referral suggest the importance of early action so subsequent quality care markers may be met.

In addition to the low rheumatologist referral rate found in our study, only 32.5% of patients received a DMARD prescription within 1 year of their index date. This finding aligned with prior studies, which have reported rates of less than 50% for patients receiving DMARD treatment within 6 to 12 months of symptom onset.^5,7^ Our study indicates that receiving DMARDs with or without hepatitis B screening is associated with greater odds of meeting later quality care markers, such as radiographs, annual laboratory testing, annual physical examination, and PT, OT, or hand surgery referral. This may be because of the association of DMARD prescription with treatment adherence; a study by Zanetti et al^22^ noted that early DMARD prescription was associated with patient adherence scores. By ensuring early DMARD treatment, long-term outcomes for patients with RA may be improved through direct disease activity and the downstream effects of meeting additional quality care markers.

Individual characteristics of patients who met quality care markers for RA varied. Women had greater odds of meeting all quality care markers except receiving DMARDs with hepatitis B screening. This may be because of the higher prevalence of RA in women.^28^ Our population comprised 74.1% women. Odds of meeting quality care markers also varied by income level. A median family income below $45 200 was associated with decreased odds of receiving a rheumatologist referral, annual physical examination, or annual laboratory testing and greater odds of receiving DMARDs with appropriate hepatitis B screening, radiographs, or PT, OT, or hand surgery referral. Some of these results align with previous literature. A study in Korea investigated the prevalence and socioeconomic status of patients with RA and found delayed diagnosis was associated with lower income.^29^ Furthermore, a Swedish study found fear-avoidance behaviors in patients with RA correlated with below-national average income levels.^30^ Although this may explain why some quality care markers were less likely to be reached in patients with lower incomes, our study could not account for all potential confounders, such as race and ethnicity.

Meeting quality care markers varied by insurance plan. Noncomprehensive insurance (preferred provider organization, point of service, health maintenance organization, or other types) was associated with greater odds of receiving a rheumatologist referral, DMARDs with hepatitis B screening, annual physical examination, and annual laboratory testing compared with comprehensive insurance. Medicare insurance had lower odds of meeting all quality care markers except hand radiographs or a PT, OT, or hand surgery referral. This finding aligned with previous research, which found that patients with Medicare who were aged 18 to 64 years were less likely to visit a rheumatologist or receive DMARDs and faced additional challenges leading to lower general health and health-related quality of life.^31^ These results may, however, be limited by discrepancies in the distribution of patients. Patients with Medicare were 0.4% of the total cohort and comprehensive insurance users made up 2.2% of the cohort.

Because the age range was 18 to 64 years, patients with Medicare in our cohort qualify for disability benefits. Individuals qualifying for disability often have high rates of chronic illness, multiple conditions, and increased social risk factors, resulting in more severe illness.^32,33^ Our findings also showed that increased comorbidities based on the RDCI, Elixhauser Comorbidity Index, and common RA comorbidities were associated with a lower likelihood of receiving a rheumatologist referral, hand radiographs, or annual physical examination. These findings highlight a lower quality of care standard for patients with multiple and severe conditions. Because of the complexity of treating RA with comorbidities, it may be beneficial to lessen the time it takes for these patients to receive a rheumatologist referral compared with the general RA population. Studies called for a shift from a rheumatologic point of view toward patient-centered care with integrated management of comorbidities.^34^ This approach focuses on treating select comorbidities that require the most active care while considering individual patient preferences and needs. Prioritizing the principal care marker—a rheumatology referral—should help achieve downstream benefits and improve additional quality care markers.

### Strengths and Limitations

This study has limitations. Claims data are unable to fully capture the clinical complexity of RA treatment and decision-making. Use of claims data also introduces the potential for a sampling bias resulting from incorrect coding and misclassification of treatments. Furthermore, changes to the insurance coverage throughout the study period may confound results. To increase the accuracy of using claims data to interpret RA diagnosis, we included a 1-year preindex and postindex visit period during which patients were continuously enrolled in insurance plans to ensure no earlier diagnosis and assess quality care markers. Several variables are not contained in the database, including race and ethnicity or educational level. These variables may confound model outcomes. Our study was strengthened by a large sample size of 581 770 patients. The database is primarily composed of claims from commercial insurers; although this represents the majority of insured patients,^35^ the cohort may not be generalizable to publicly insured populations. In addition, our study spanned several years with influential policy implementation, such as the 2014 Affordable Care Act expansion; however, previous studies have found little or no change in quality over long-term follow-up for ambulatory care following this implementation.^36^ An additional limitation of the study is a follow-up period of 1 year. However, adjusting the follow-up period to 2 years did not statistically change the results (eTable 3 in Supplement 1). Finally, we recognize the lack of causality as a limitation of this study. The sequential modeling lends itself to evaluating a realistic, yet hypothetical, care cascade for patients with RA. Although this should not be interpreted as a causal relationship, we elected this particular care cascade for its commonality.

## Conclusions

This study proposed a sequential pathway for RA quality care markers and identified patient characteristics that show variation in meeting these markers. We found an association between rheumatologist referral and receiving DMARDs on meeting other quality care markers. We also found that men, patients with Medicare, and patients with increased comorbidities were generally less likely to meet quality care markers. Although this study is based on a hypothetical care model, it supports prior research that has found variable quality of RA care and indicates specific patient populations that can be targeted for improvement. In addition, this study supports the Arthritis Foundation guidelines and the importance of early diagnosis and care for patients with RA.

## Notes

Supplement 1. **eTable 1.** List of Dependent and Independent Variables and Their Respective Codes **eTable 2.** Odds Ratios and 95% CIs for the Terms in the 6 Logistic Regression Models (1 Year) **eTable 3.** Odds Ratios and 95% CIs for the Terms in the 6 Logistic Regression Models (2 Years)

Supplement 2.
Data Sharing Statement
