# Supplementary material for: Factors Associated With Quality Care Among Adults With Rheumatoid Arthritis
Source: JAMA Netw Open. 2022 Dec 12;5(12):e2246299. doi: 10.1001/jamanetworkopen.2022.46299 (PMC9856345; doi:10.1001/jamanetworkopen.2022.46299)
Supplement: Supplement 1. — eTable 1. List of Dependent and Independent Variables and Their Respective Codes eTable 2. Odds Ratios and 95% CIs for the Terms in the 6 Logistic Regression Models (1 Year) eTable 3. Odds Ratios and 95% CIs for the Terms in the 6 Logistic Regression Models (2 Years) [file jamanetwopen-e2246299-s001.pdf]

Supplementary Online Content

Seyferth AV, Cichocki MN, Wang C-W, et al. Factors associated with quality care among adults with rheumatoid arthritis. *JAMA Netw Open*. 2022;5(12):e2246299. doi:10.1001/jamanetworkopen.2022.46299

eTable 1. List of Dependent and Independent Variables and Their Respective Codes

eTable 2. Odds Ratios and 95% CIs for the Terms in the 6 Logistic Regression Models (1 Year)

eTable 3. Odds Ratios and 95% CIs for the Terms in the 6 Logistic Regression Models (2 Years)

This supplementary material has been provided by the authors to give readers additional information about their work.

© 2022 Seyferth AV et al. *JAMA Network Open.*

eTable 1. List of dependent and independent variables and their respective codes.

Variable	Algorithm used to identify	Codes
**Inclusion criteria**		
Rheumatoid arthritis	Identify first encounter with RA using ICD9/10 codes. Included a 1-year pre-index look-back period (or to the earliest date of the claim for patients in 2009) to ensure no prior RA diagnosis and a 1-year post-index date follow-up.	ICD-9: 714.xx.<br>ICD-10: M05.XX, M06.XX
**Exclusion criteria**		
Psoriatic arthritis	Had encounter during observation time with corresponding ICD codes	ICD-9: 696.0X<br>ICD-10: L40.5X
Acute viral polyarthritis	Had encounter during observation time with corresponding ICD codes	ICD-9: 716.5X (079.0-079.99)<br>ICD-10: M13.0X
Gout	Had encounter during observation time with corresponding ICD codes	ICD-9: 274.0X, 274.1X, 274.8X, 274.9X<br>ICD-10: M10.X, M10.1X, M10.2X, M10.3X, M10.4X, M10.9X
Calcium pyrophosphate deposition disease	Had encounter during observation time with corresponding ICD codes	ICD-9: 712.1X, 712.2X, 712.3X<br>ICD-10: M11.1X, M11.2X
System lupus erythematosus	Had encounter during observation time with corresponding ICD codes	ICD-9: 710.XX<br>ICD-10: M32.XX
**Comorbidity***		
Gastrointestinal	Had ICD codes within 3 years before index date	ICD-9: 530.2X, 530.4X, 530.8X, 531.0X-531.2X, 531.4X-531.6X, 532.0X-532.2X, 532.4X-532.6X, 533.0X-533.2X, 533.4X-533.6X, 534.0X- 534.2X, 534.4X- 534.6X, 540.0X, 540.1X, 540.9X, 562.0X, 562.1X, 569.8X<br>ICD-10: K22.1X, K22.3X, K25.0X-K25.2X, K25.4X-K25.6X, K26.0X-K26.2X, K26.4X-K26.6X, K27.0X-K27.2X, K27.4X-K27.6X, K28.0X-K28.2X, K28.4X-K28.6X, K35.2X, K35.3X, K57.0X-K57.5X, K57.8X, K57.9X, K63.1X<br>Diverticulosis/Diverticulitis need evidence of confirmatory surgery via CPT<br>ICD-9: 562.0X, 562.1 need with CPT code 44602, 44603, 44120, 44121, 44125, 44130, 44202, or 44203<br>Ischemic colitis needs evidence of confirmatory surgery via CPT<br>ICD-9: 557.0X, 557.1X, 557.9X<br>ICD-10: K55.0X, K55.1X, K55.3X, K55.8X, K55.9X<br>with CPT code 44604, 44605, 44140, 44145, 44204, or 44205
Heart Disease	Had ICD codes within 3 years before index	ICD-9: 394.XX-396.XX, 402.XX, 404.XX, 410.XX-414.XX, 425.XX-428.XX, 424.0X-424.3X

© 2022 Seyferth AV et al. *JAMA Network Open.*

	date	ICD-10: I05.0X-I05.2X, I05.8X, I05.9X, I06.0X-I06.2X, I06.8X, I06.9X, I08.0X, I08.8X, I08.9X, I11.0X, I11.9X, I13.0X-I13.2X, I20.0X, I20.1X, I20.8X, I20.9X, I21.0X-I21.4X, I22.0X-I22.2X, I22.8X, I22.9X, I24.0X, I24.1X, I24.8X, I24.9X, I25.1X-I25.9X, I34.0X-I34.2X, I34.8X, I34.9X, I35.0X-I35.2X, I35.8X, I35.9X, I36.0X-I36.2X, I36.8X, I36.9X, I37.0X-I37.2X, I37.8X, I37.9X, I42.0X-I42.9X, I43.XX, I44.0X- I44.7X, I45.0X-I45.9X, I46.2X, I46.8X, I46.9X, I47.0X-I47.2X, I47.9X, I48.0X, I48.1X, I49.0X-I49.5X, I49.8X, I49.9X, I50.1X-I50.4X, I50.9X, R00.1X, R09.8X
Diabetes	Had ICD codes within 3 years before index date	ICD-9: 249.XX, 250.XX<br>ICD-10: E10.1X-E10.6X, E10.8X, E10.9X, E11.0X, E11.2X-E11.6X, E11.8X, E11.9X
**Model Outcomes**+		
DMARDs	1-year post-index date	We used the National Drug Code numbers to identify bDMARDs, cDMARDs, and tsDMARDs.
Hepatitis B Screening	1-year post-index date	CPT/Procedure: 86704, 86705, 86706, 86707, 87340, 87341, 87350
Hand X-Ray	1-year post-index date	CPT/Procedure: 73090, 73100, 73110, 73115, 73120, 73130, 73140
Annual physical exam	1-year post-index date	CPT/Procedure: 99385, 99386, 99395, 99396, 92002, 92004, 92012, 92014
Annual lab work	1-year post-index date	CPT/Procedure:85652, 86140, 86430, 80048
PT/OT	1-year post-index date	PT CPT/Procedure: 97001, 97161, 97162, 97163, 97002, 97164<br>OT CPT/Procedure: 97003, 91765, 97166, 97167, 97004, 97168

© 2022 Seyferth AV et al. *JAMA Network Open.*

eTable 2: Odds ratios and 95% CIs for the terms in the 6 logistic regression models (1 year)

Variables/ Model	1.Referral to a rheumatologist	2.DMARDs/ HepB screening	3.Hand X-ray	4.Annual physician exam	5.Annual lab work	6.Referral to hand/OT/PT
Sex Male (ref: Female)	0.72 (0.72-0.73)	1.14 (1.12-1.16)	0.86 (0.84-0.88)	0.78 (0.76-0.81)	0.89 (0.88-0.90)	0.86 (0.84-0.89)
Age: 25-34 (ref: 18-24)	1.16 (1.12-1.19)	0.84 (0.81-0.87)	1.16 (1.09-1.23)	0.74 (0.69-0.80)	1.04 (1.01-1.08)	0.81 (0.74-0.88)
Age: 35-44 (ref: 18-24)	1.26 (1.22-1.29)	0.77 (0.75-0.80)	1.28 (1.20-1.35)	0.86 (0.80-0.93)	1.10 (1.06-1.13)	0.82 (0.75-0.88)
Age: 45-54 (ref: 18-24)	1.33 (1.29-1.37)	0.71 (0.69-0.74)	1.34 (1.27-1.42)	0.96 (0.90-1.03)	1.07 (1.04-1.10)	0.97 (0.9-1.050)
Age: 55-64 (ref: 18-24)	1.43 (1.39-1.47)	0.67 (0.65-0.69)	1.33 (1.26-1.41)	1.14 (1.06-1.22)	0.96 (0.93-0.99)	1.10 (1.02-1.19)
Country region: North Central (ref: Northeast)	1.07 (1.05-1.09)	0.46 (0.45-0.47)	1.47 (1.42-1.51)	0.79 (0.76-0.83)	0.82 (0.81-0.83)	1.33 (1.27-1.39)
Country region: South (ref: Northeast)	1.97 (1.94-2.00)	0.72 (0.70-0.73)	1.54 (1.50-1.59)	0.75 (0.72-0.77)	0.97 (0.96-0.99)	1.27 (1.22-1.32)
Country region: West (ref: Northeast)	1.12 (1.10-1.14)	0.59 (0.58-0.60)	1.30 (1.26-1.34)	0.85 (0.81-0.88)	0.87 (0.85-0.88)	1.37 (1.30-1.43)
Median household income: Above 45,200 (ref: Below 45,200)	1.19 (1.17-1.21)	0.96 (0.95-0.98)	0.84 (0.82-0.86)	1.21 (1.16-1.26)	1.08 (1.06-1.10)	0.93 (0.90-0.97)
Insurance plan: Comprehensive (ref: Others)	0.69 (0.67-0.72)	0.95 (0.91-0.99)	1.00 (0.94-1.07)	0.69 (0.62-0.77)	0.76 (0.73-0.79)	1.11 (1.02-1.21)
Insurance source: Medicare (ref: Commercial)	0.77 (0.71-0.84)	0.62 (0.57-0.67)	1.06 (0.91-1.22)	0.63 (0.48-0.82)	0.26 (0.24-0.29)	1.09 (0.90-1.30)
RDCI: 1-2 (ref: 0)	0.88 (0.87-0.90)	1.07 (1.05-1.08)	0.99 (0.96-1.01)	0.91 (0.88-0.94)	1.05 (1.04-1.07)	1.03 (0.99-1.07)
RDCI: >2 (ref: 0)	0.88 (0.86-0.90)	1.07 (1.05-1.10)	0.99 (0.96-1.02)	0.77 (0.73-0.80)	1.07 (1.05-1.09)	1.06 (1.01-1.11)

© 2022 Seyferth AV et al. *JAMA Network Open.*

Elixhauser: 1-3 (ref: 0)	0.91 (0.89-0.93)	1.19 (1.16-1.21)	0.92 (0.89-0.96)	0.80 (0.76-0.84)	0.96 (0.94-0.98)	1.10 (1.05-1.15)
Elixhauser: 4-8 (ref: 0)	0.85 (0.84-0.86)	1.33 (1.31-1.35)	0.97 (0.94-0.99)	0.71 (0.69-0.74)	1.01 (0.99-1.02)	0.98 (0.95-1.02)
Elixhauser: >8 (ref: 0)	0.84 (0.83-0.86)	1.38 (1.36-1.41)	0.92 (0.89-0.94)	0.68 (0.65-0.71)	0.92 (0.91-0.94)	1.03 (0.99-1.07)
Heart Diseases: Yes (ref: Non)	0.96 (0.95-0.97)	1.07 (1.05-1.08)	0.98 (0.96-1.00)	0.90 (0.86-0.93)	1.03 (1.02-1.05)	1.04 (1.00-1.08)
Diabetes: Yes (ref: Non)	0.77 (0.76-0.78)	1.17 (1.15-1.19)	0.89 (0.87-0.91)	0.59 (0.56-0.62)	1.02 (1.00-1.03)	0.82 (0.79-0.85)
GI_Bleeds: Yes (ref: Non)	1.00 (0.99-1.01)	1.14 (1.12-1.15)	1.02 (1.00-1.04)	0.98 (0.96-1.02)	1.07 (1.06-1.09)	1.08 (1.04-1.11)
Referral to a rheumatologist: Yes (ref: Non)	-	0.24 (0.24-0.25)	2.35 (2.30-2.39)	0.99 (0.96-1.02)	1.93 (1.91-1.95)	0.92 (0.89-0.95)
DMARDs/ HepB screening: no DMARDs/ yes HepB screening (ref: no DMARDs/ no HepB screening)	-	-	3.63 (3.49-3.78)	0.96 (0.88-1.05)	4.23 (4.06-4.40)	0.88 (0.80-0.96)
DMARDs/ HepB screening: yes DMARDs/ no HepB screening (ref: no DMARDs/ no HepB screening)	-	-	1.61 (1.58-1.64)	1.95 (1.90-2.01)	2.19 (2.16-2.22)	1.42 (1.37-1.46)
DMARDs/ HepB screening: yes DMARDs/ yes HepB screening (ref: no DMARDs/ no HepB screening)	-	-	5.74 (5.55-5.94)	2.16 (2.03-2.29)	8.92 (8.48-9.37)	1.46 (1.36-1.56)
Hand X-ray: Yes (ref: Non)	-	-	-	1.07 (1.02-1.12)	2.02 (1.97-2.06)	2.41 (2.33-2.50)
Annual physician exam: Yes (ref: Non)	-	-	-	-	0.95 (0.92-0.98)	0.96 (0.89-1.02)
Annual lab work: Yes (ref: Non)	-	-	-	-	-	0.88 (0.86-0.91)

© 2022 Seyferth AV et al. *JAMA Network Open.*

eTable 3: Odds ratios and 95% CIs for the terms in the 6 logistic regression models (2 years)

Referral to hand/OT/PT	1.Referral to a rheumatologist	2.DMARDs/ HepB screening	3.Hand X-ray	4.Annual physician exam	5.Annual lab work	6.Referral to hand/OT/PT
Sex Male (ref: Female)	0.72 (0.72-0.73)	1.15 (1.14-1.17)	0.85 (0.83-0.87)	0.78 (0.76-0.80)	0.92 (0.91-0.93)	0.82 (0.80-0.85)
Age: 25-34 (ref: 18-24)	1.16 (1.12-1.19)	0.85 (0.82-0.88)	1.15 (1.08-1.21)	0.76 (0.71-0.82)	1.04 (1.00-1.07)	0.83 (0.77-0.90)
Age: 35-44 (ref: 18-24)	1.26 (1.22-1.29)	0.78 (0.76-0.81)	1.27 (1.20-1.34)	0.92 (0.86-0.98)	1.08 (1.04-1.11)	0.84 (0.78-0.90)
Age: 45-54 (ref: 18-24)	1.33 (1.29-1.37)	0.73 (0.7-0.75)	1.34 (1.27-1.41)	1.02 (0.96-1.09)	1.04 (1.01-1.07)	1.01 (0.94-1.08)
Age: 55-64 (ref: 18-24)	1.43 (1.39-1.47)	0.69 (0.67-0.71)	1.34 (1.27-1.41)	1.17 (1.10-1.25)	0.96 (0.93-0.98)	1.15 (1.07-1.24)
Country region: North Central (ref: Northeast)	1.07 (1.05-1.09)	0.47 (0.46-0.48)	1.45 (1.40-1.49)	0.77 (0.75-0.80)	0.80 (0.79-0.81)	1.27 (1.22-1.33)
Country region: South (ref: Northeast)	1.97 (1.94-2.00)	0.72 (0.70-0.73)	1.49 (1.46-1.53)	0.70 (0.68-0.73)	0.99 (0.97-1.00)	1.19 (1.15-1.24)
Country region: West (ref: Northeast)	1.12 (1.10-1.14)	0.59 (0.58-0.60)	1.29 (1.25-1.33)	0.86 (0.83-0.90)	0.84 (0.83-0.86)	1.36 (1.31-1.42)
Median household income: Above 45,200 (ref: Below 45,200)	1.19 (1.17-1.21)	0.97 (0.96-0.99)	0.84 (0.82-0.86)	1.22 (1.17-1.26)	1.09 (1.08-1.11)	0.94 (0.90-0.97)
Insurance plan: Comprehensive (ref: Others)	0.69 (0.67-0.72)	0.95 (0.91-0.99)	0.98 (0.92-1.04)	0.74 (0.67-0.81)	0.75 (0.72-0.78)	1.08 (1.00-1.18)
Insurance source: Medicare (ref: Commercial)	0.77 (0.71-0.84)	0.61 (0.56-0.66)	1.03 (0.90-1.18)	0.59 (0.46-0.75)	0.20 (0.18-0.22)	1.08 (0.92-1.28)
RDCI: 1-2 (ref: 0)	0.88 (0.87-0.90)	1.05 (1.04-1.07)	1.02 (0.99-1.04)	0.96 (0.93-0.98)	1.01 (1.00-1.03)	1.08 (1.05-1.12)
RDCI: >2 (ref: 0)	0.88 (0.86-0.90)	1.03 (1.01-1.05)	1.03 (1.00-1.06)	0.83 (0.79-0.86)	0.98 (0.96-1.00)	1.17 (1.13-1.23)
Elixhauser: 1-3 (ref: 0)	0.91 (0.89-0.93)	1.20 (1.18-1.23)	0.90 (0.88-0.93)	0.79 (0.76-0.83)	1.00 (0.98-1.02)	1.06 (1.01-1.11)

© 2022 Seyferth AV et al. *JAMA Network Open.*

Elixhauser: 4-8 (ref: 0)	0.85 (0.84-0.86)	1.33 (1.31-1.35)	0.95 (0.93-0.97)	0.72 (0.69-0.74)	1.03 (1.02-1.05)	0.97 (0.94-1.01)
Elixhauser: >8 (ref: 0)	0.84 (0.83-0.86)	1.39 (1.37-1.42)	0.90 (0.88-0.92)	0.68 (0.65-0.71)	0.97 (0.95-0.98)	1.00 (0.96-1.04)
Heart Diseases: Yes (ref: Non)	0.96 (0.95-0.97)	1.06 (1.05-1.08)	0.99 (0.97-1.02)	0.92 (0.88-0.95)	1.02 (1.00-1.03)	1.04 (1.01-1.08)
Diabetes: Yes (ref: Non)	0.77 (0.76-0.78)	1.18 (1.16-1.20)	0.89 (0.86-0.91)	0.60 (0.57-0.62)	1.04 (1.02-1.05)	0.82 (0.79-0.85)
GI_Bleeds: Yes (ref: Non)	1.00 (0.99-1.01)	1.11 (1.10-1.12)	1.06 (1.04-1.08)	1.05 (1.02-1.08)	1.02 (1.00-1.03)	1.11 (1.08-1.14)
Referral to a rheumatologist: Yes (ref: Non)	-	0.25 (0.24-0.25)	2.23 (2.19-2.28)	1.06 (1.04-1.09)	1.88 (1.86-1.90)	1.01 (0.99-1.04)
DMARDs/ HepB screening: no DMARDs/ yes HepB screening (ref: no DMARDs/ no HepB screening)	-	-	3.62 (3.48-3.76)	1.17 (1.09-1.27)	4.48 (4.32-4.66)	0.98 (0.90-1.06)
DMARDs/ HepB screening: yes DMARDs/ no HepB screening (ref: no DMARDs/ no HepB screening)	-	-	1.95 (1.91-1.99)	2.23 (2.17-2.29)	1.84 (1.82-1.87)	1.61 (1.56-1.66)
DMARDs/ HepB screening: yes DMARDs/ yes HepB screening (ref: no DMARDs/ no HepB screening)	-	-	6.14 (5.95-6.33)	2.62 (2.50-2.76)	8.87 (8.48-9.28)	1.72 (1.63-1.82)
Hand X-ray: Yes (ref: Non)	-	-	-	1.26 (1.22-1.31)	2.17 (2.12-2.21)	2.37 (2.30-2.44)
Annual physician exam: Yes (ref: Non)	-	-	-	-	1.05 (1.02-1.07)	1.17 (1.12-1.23)
Annual lab work: Yes (ref: Non)	-	-	-	-	-	0.94 (0.92-0.97)

© 2022 Seyferth AV et al. *JAMA Network Open.*
