# Supplementary material for: Factors Associated With Quality Care Among Adults With Rheumatoid Arthritis
Source: JAMA Netw Open. 2022 Dec 12;5(12):e2246299. doi: 10.1001/jamanetworkopen.2022.46299 (PMC9856345; doi:10.1001/jamanetworkopen.2022.46299)
Supplement: Supplement 2. — Data Sharing Statement [file jamanetwopen-e2246299-s002.pdf]

Data Sharing Statement

Seyferth. Factors Associated With Quality Care Among Adults With Rheumatoid Arthritis. *JAMA Netw Open*. Published December 12, 2022. doi:10.1001/jamanetworkopen.2022.46299

Data
Data available: Yes
Data types: Data (not involving human participants), Data dictionary
How to access data: Data dictionary of ICD/CPT codes used can be found in Supplemental Table 1. Additional data can be requested by emailing: aseyf@med.umich.edu
When available: With publication

Supporting Documents
Document types: None

Additional Information
Who can access the data: Anyone requesting the data
Types of analyses: For any purpose
Mechanisms of data availability: With investigator support
